# Supplementary material for: C-COMPASS: a user-friendly neural network tool profiles cell compartments at protein and lipid levels
Source: Nat Methods. 2025 Dec 4;23(1):118–30. doi: 10.1038/s41592-025-02880-3 (PMC12791020; doi:10.1038/s41592-025-02880-3)
Supplement: Supplementary file 1 — Procedural suggestion for generating a custom marker list [file 41592_2025_2880_MOESM1_ESM.pdf]

nature methods

Supplementary information https://doi.org/10.1038/s41592-025-02880-3

C-COMPASS: a user-friendly neural network tool profiles cell compartments at protein and lipid levels

In the format provided by the authors and unedited

Inventory of Supplementary information

- Procedural suggestion for generating a custom marker list

Procedural suggestion for generating a custom marker list

In certain cases, it may be necessary to generate a custom list of spatial marker proteins, particularly when reference data for specific tissues, organisms, or biological conditions is unavailable. However, it is possible to derive marker proteins that reflect compartment-specific profile patterns directly from the dataset itself. To do so, we recommend the following pre-processing steps on the gradient data: 1) Impute missing values with zero. 2) Apply MinMax scaling to normalize all protein profiles to a range between 0 and 1.

Next, perform hierarchical clustering across the protein dimensions (rows) only, without clustering the fraction dimension (columns). After clustering, identify protein clusters that are enriched for annotations associated with specific organelles or compartments. We recommend using Gene Ontology Cellular Component (GOCC) annotations for this purpose. This enrichment can be assessed manually or via algorithmic approaches that identify clusters with the strongest enrichment for the compartments of interest. If performed manually, we recommend prioritizing clusters with distinctive profile patterns over large cluster sizes.

The resulting set of proteins can then be compiled into a marker list, with each protein annotated according to the enriched compartment. To be used in C-COMPASS, this list must contain at least two columns: one serving as an identifier (e.g. gene names), and one indicating the assigned compartment annotation. A comprehensive list of marker proteins used in our experiments can be found in the supplementary material.

Below, we provide a list of publications in which this strategy has been successfully applied to derive compartment-specific marker proteins:

Klingelhuber, F. et al. A spatiotemporal proteomic map of human adipogenesis. *Nat Metab* **6**, 861-879 (2024)

Krahmer, N. et al. Organellar Proteomics and Phospho-Proteomics Reveal Subcellular Reorganization in Diet-Induced Hepatic Steatosis. *Dev Cell* **47**, 205-221 e207 (2018)
